# Clinical relevance of microRNA miR-21, miR-31, miR-92a, miR-101, miR-106a and miR-145 in colorectal cancer

**DOI:** 10.1186/1471-2407-12-505

**Published:** 2012-11-02

**Authors:** Kristina Schee, Kjetil Boye, Torveig Weum Abrahamsen, Øystein Fodstad, Kjersti Flatmark

**Affiliations:** 1 Departments of Tumor Biology, The Norwegian Radium Hospital, Oslo University Hospital, N-0310, Oslo, Norway; 2 Departments of Oncology, The Norwegian Radium Hospital, Oslo University Hospital, N-0310, Oslo, Norway; 3 Departments of Gastrointestinal Surgery, The Norwegian Radium Hospital, Oslo University Hospital, N-0310, Oslo, Norway

**Keywords:** MiRNA, Colorectal cancer, Prognostic biomarker

## Abstract

**Background:**

MicroRNAs (miRNAs) regulate gene expression by binding to mRNA, and can function as oncogenes or tumor suppressors depending on the target. In this study, using qRT-PCR, we examined the expression of six miRNAs (miR-21, miR-31, miR-92a, miR-101, miR-106a and miR-145) in tumors from 193 prospectively recruited patients with colorectal cancer, and associations with clinicopathological parameters and patient outcome were analyzed. The miRNAs were chosen based on previous studies for their biomarker potential and suggested biological relevance in colorectal cancer.

**Methods:**

The miRNA expression was examined by qRT-PCR. Associations between miRNA expression and clinicopathological variables were explored using Mann–Whitney U and Kruskal-Wallis test while survival was estimated using the Kaplan-Meier method and compared using the log-rank test.

**Results:**

MiR-101 was hardly expressed in the tumor samples, while for the other miRNAs, variable expression levels and expression ranges were observed, with miR-21 being most abundantly expressed relative to the reference (RNU44). In our study cohort, major clinical significance was demonstrated only for miR-31, as high expression was associated with advanced tumor stage and poor differentiation. No significant associations were found between expression of the investigated miRNAs and metastasis-free or overall survival.

**Conclusions:**

Investigating the expression of six miRNAs previously identified as candidate biomarkers in colorectal cancer, few clinically relevant associations were detected in our patient cohort. Our results emphasize the importance of validating potential tumor markers in independent patient cohorts, and indicate that the role of miRNAs as colorectal cancer biomarkers is still undetermined.

## Background

MicroRNAs (miRNAs) are a class of small, non-coding RNAs (19–22 nucleotides) that function as posttranscriptional gene regulators by binding to the 3’UTR of mRNA, and one miRNA may potentially down-regulate multiple mRNA targets. More than 1500 human miRNAs are currently annotated in the miRBase [1], and it has been predicted that as many as 30% of protein-encoding genes may be regulated by miRNAs [2]. The discovery that miRNAs may function as oncogenes or tumor suppressors depending on the target mRNA, has instigated intensive research to determine the role of these molecules in cancer. MiRNAs are chemically very stable, and can be detected by a range of high-throughput detection methods in tissue, serum and plasma as well as in urine and feces, and are for these reasons considered to have great potential as cancer biomarkers.

In colorectal cancer (CRC), treatment decisions are still based essentially on anatomical extent of disease at diagnosis, and the search for better biomarkers is warranted. Several miRNAs with potential biological and clinical relevance have been identified and are being explored as diagnostic, prognostic and predictive biomarkers [3-6]. Based on previous studies and our recent review of this topic, six candidate miRNAs, miR-21, miR-31, miR-92a, miR-101, miR-106a and miR-145 (Table 1), were chosen for analysis in a cohort of 193 prospectively recruited patients receiving curative surgery for CRC [7-13]. Expression of the miRNA was determined by qRT-PCR and associations with clinicopathological parameters and outcome were analyzed.

**Table 1:** Mature sequence, miRBase accession number and proposed clinical relevance for the six chosen miRNAs

**miRNA name**	**Mature miRNA sequence**	**miRBase Accession number**	**Proposed clinical relevance**	**Comment**	**Reference**
hsa-miR-21	UAGCUUAUCAGACUGAUGUUGA	MIMAT0000076	Overall survival	High expression associated with poor OS	[14]
hsa-miR-31	AGGCAAGAUGCUGGCAUAGCU	MIMAT0000089	Tumor stage/ differentiation	High expression associated with advanced tumor stage and poorly differentiated tumors	[10]
hsa-miR-92a	UAUUGCACUUGUCCCGGCCUGU	MIMAT0000092	Plasma marker	Elevated levels as a possible diagnostic marker	[13]
hsa-miR-101	UACAGUACUGUGAUAACUGAA	MIMAT0000099	Increased invasiveness	Decreased expression associated with invasiveness	[8]
hsa-miR-106a	AAAAGUGCUUACAGUGCAGGUAG	MIMAT0000103	Disease free and overall survival	Down-regulation associated with poor disease free and overall survival.	[7]
hsa-miR-145	GUCCAGUUUUCCCAGGAAUCCCU	MIMAT0000437	Tumor size	Low expression associated with large tumor size	[9]

## Methods

### Patient cohort

316 patients, recruited from five hospitals in the Oslo region between the year 1998 and 2000 [15], were prospectively included in the study at the time of primary surgery for assumed or verified colorectal cancer. The study was approved by the Regional Ethics Committee (Health Region II, Norway) and informed consent was obtained from the patients. At surgery, resected specimens were routinely processed for histopathological assessment and additional tumor tissue was sampled and snap-frozen in liquid nitrogen. A number of cases were excluded from statistical analysis for the following reasons: not invasive cancer (25), histology other than adenocarcinoma (5), distant metastasis at the time of surgery (34, tissue samples not available), preoperative chemoradiotherapy (2), inadequate surgical margins (7), unknown stage of disease (1), freshly frozen tissue samples not obtainable (46), and high Ct-values (>37; n=3). The study population thus consisted of 193 patients in TNM stage I-III (Table 2). Follow-up data was obtained from the participating hospitals and from the general practitioners (for the patients not attending scheduled controls). Metastasis was verified by radiological examination and survival data was obtained from the National Registry of Norway and updated by October 1^st^ 2008 with the cause of death registered and classified as death from colorectal cancer, death of other cause or death of unknown cause.

**Table 2:** Median expression levels of the six selected miRNAs and associations with clinicopathological data

	**Number (percent)**	**miR-21**	**miR-31**	**miR-92a**	**miR-101**	**miR-106a**	**miR-145**
**Gender**							
Female	81 (42)	7.78	0.07	1.64	0.02	1.16	0.43
Male	112 (58)	7.53	0.03	2.12	0.02	0.93	0.48
*p-value*		*0.45*	*0.14*	*0.52*	*0.78*	*0.43*	*0.99*
**TNM**							
I	35 (18)	5.22	0.02	2.41	0.02	1.24	0.34
II	97 (50)	7.67	0.06	2.09	0.02	1.10	0.48
III	61 (32)	7.78	0.07	1.59	0.02	0.85	0.51
*p-value*		*0.23*	*0.02*	*0.80*	*0.86*	*0.54*	*0.30*
**pT**							
1	4 (2)	7.90	0.02	2.51	0.02	1.16	0.36
2	36 (19)	5.15	0.02	2.45	0.02	1.26	0.36
3	133 (69)	7.67	0.05	1.74	0.02	0.88	0.46
4	20 (10)	8.50	0.14	2.58	0.02	1.33	0.58
*p-value*		*0.37*	*0.004*	*0.61*	*0.76*	*0.52*	*0.70*
**pN**							
0	132 (68)	7.53	0.04	2.12	0.02	1.15	0.44
1	39 (20)	7.67	0.05	1.73	0.02	0.89	0.46
2	22 (11)	8.47	0.09	1.39	0.02	0.82	0.59
*p-value*		*0.82*	*0.31*	*0.60*	*0.95*	*0.54*	*0.63*
**Differentiation**							
Well	6 (3)	3.58	0.02	1.09	0.01	0.38	0.24
Intermediate	167 (87)	7.67	0.04	2.14	0.02	1.16	0.45
Poor	20 (10)	6.83	0.20	0.95	0.02	0.70	0.69
*p-value*		*0.28*	*0.001*	*0.003*	*0.33*	*0.01*	*0.12*
**Tumor localization**							
Colon	129 (67)	7.52	0.07	1.62	0.02	0.87	0.43
Rectum	64 (33)	7.77	0.02	2.58	0.02	1.27	0.67
*p-value*		*0.50*	*0.02*	*0.05*	*0.32*	*0.05*	*0.08*
**Lymphocyte infiltration**							
High	26 (14)	7.93	0.09	1.96	0.02	0.88	0.63
Intermediate	125 (65)	7.78	0.03	2.14	0.02	1.01	0.51
Low	40 (21)	6.91	0.08	1.46	0.02	1.04	0.33
*p-value*		*0.47*	*0.19*	*0.49*	*0.82*	*0.37*	*0.14*
**Vascular invasion**							
Present	38 (20)	7.73	0.07	1.54	0.02	1.17	0.59
Absent	155 (80)	7.36	0.04	2.01	0.02	0.98	0.43
*p-value*		*0.30*	*0.23*	*0.43*	*0.27*	*0.94*	*0.05*
**Perineural invasion**							
Present	16 (8)	8.60	0.16	1.68	0.02	1.05	0.35
Absent	177 (92)	7.54	0.04	1.95	0.02	0.98	0.48
*p-value*		*0.42*	*0.43*	*0.60*	*0.29*	*0.83*	*0.49*
Perinodal growth*							
Present	38 (62)	8.09	0.11	1.46	0.02	0.81	0.52
Absent	23 (38)	7.36	0.03	2.01	0.02	1.10	0.36
*p-value*		*0.77*	*0.36*	*0.30*	*1.00*	*0.18*	*0.49*

*Associations between miRNA expression and clinicopathological variables were explored using Mann–Whitney or Kruskal-Wallis test as appropriate; p-values are given in italic. Total number of patients included in the analyses was 193, median age was 73 years.

** Perinodal growth was only assessed in the lymph node positive patients.

### MiRNA selection

MiRNA selection was based on previous studies and our literature review [11], identifying miRNA with proposed clinical relevance in CRC, including published articles leading up to the year 2009. We wished to examine selected miRNAs in our CRC cohort and their relevance with clinicopathological data and outcome parameters (Table 2). The following six miRNAs were chosen for analysis; miR-21, miR-31, miR-92a, miR-101, miR-106a and miR-145 [7-13].

### Sample preparation and RNA isolation

Biopsies were sampled and snap frozen in liquid nitrogen and stored at −80°C. The biopsies were sectioned using a cryostat microtome and hematoxylin-eosin stained slides were evaluated for tumor content by a pathologist (median tumor content in the samples was 50%, range 30 - 80%). The tumor tissue was sliced into 10 μm sections using a cryostat microtome, aliquoted into 1.5 ml Micro tubes (Sarstedt, Nümbrecht, Germany) and stored at −80°C. RNA was isolated from the tumor tissue using TriReagent (Ambion Inc, TX) according to the manufacturer’s protocol and the total RNA concentration was measured by Nanodrop (ND-1000).

### qRT-PCR

Total RNA from 196 patients was used to reversely transcribe miRNAs using TaqMan MicroRNA assays (Applied Biosystems, Foster City, CA). Each reverse transcriptase reaction contained 10 ng of total RNA (5μl), 0.15 μl dNTP (100 mM total), 1.0 μl Multiscribe RT enzyme (50 U/μl), 1.5 μl 10X RT buffer, 0.19 μl RNase Inhibitor (20 U/μl) , 4.16 μl nuclease free water (Sigma-Aldrich, Ayshire, UK) and 3.0 μl 5X RT Primer. The 15 μl reaction volumes were incubated in 8-well PCR strip tubes (Sarstedt) in a GeneAmp PCR System 9700 thermal cycler (Applied Biosystems) as follows; 30 min at 16°C, 30 min at 42°C, 5 min at 85°C. Real-time PCR was performed using Applied Biosystems 7500 real-time PCR system. The reversely transcribed miRNAs were diluted 1:20 before adding 1.3 μl to 10 μl 2X Universal PCR Master Mix (no AmpErase UNG), 7.7 μl water and 1.0 μl 20X MicroRNA Assay. A total volume of 20 μl per reactions was incubated in 96-well MicroAmp plates (Applied Biosystems) for 10 min 95°C followed by 40 cycles of 15 sec. 95°C and 60 sec. 60°C. All samples were run in duplicates.

RNU6B and RNU44 were tested as potential reference genes and performed equally well, and RNU44 was selected for further analysis [16]. Each miRNA was normalized against RNU44 and the relative expression was calculated using 2^-dCt^ method [17].

### Statistical analysis

All statistical analyses were performed using SPSS version 18.0 (SPSS Inc., Chicago, MO) and P-values < 0.05 were considered to be statistically significant. Associations between miRNA expression and clinicopathological variables were explored using Mann–Whitney U and Kruskal-Wallis test as appropriate. Survival was estimated using the Kaplan-Meier method and compared using the log-rank test. Overall and metastasis-free survival was calculated from date of surgery until date of death or diagnosis of metastasis.

## Results

### MiRNA expression in tumor samples

**Figure 1:**
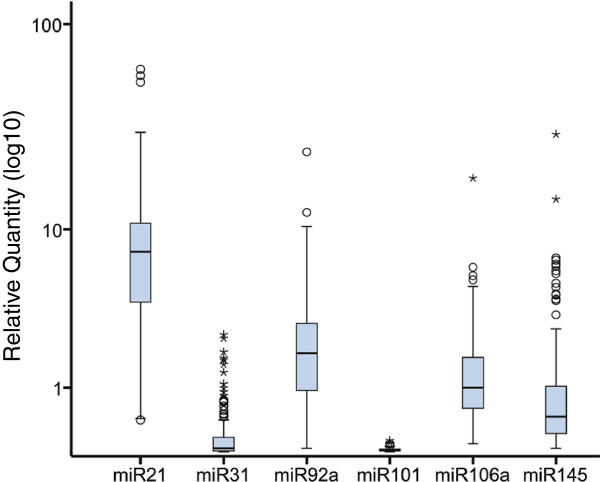
**MiRNA expression in tumor samples.** Boxplot showing the relative expression distribution of miR-21, miR-31, miR-92a, miR-101, miR-106a and miR-145. qRT-PCR was performed and Ct values for each miRNA was normalized against RNU44 and the relative expression was calculated using 2^-dCt^ method. Circles represent outliers while stars represent extreme outliers.

The most abundantly expressed miRNA relative to the reference was miR-21, and it also exhibited the widest expression range among the examined candidates (median relative expression ratio was 7.7; range 0.4-61.0). In contrast, miR-101 was hardly detectable in any of the samples (0.02; 0–0.13), and miR-31 exhibited low expression but a wider expression range (0.04; 0–2.6). The remaining three miRNAs, miR-92a (1.9; 0.04-24.4), miR-106a (1.0; 0.1-18.1), and miR-145 (0.5; 0.04-29.8) exhibited intermediate expression levels and variability between samples (Figure 1).

### MiRNA expression and associations with clinicopathological parameters

To explore the clinical significance of these findings, associations with clinicopathological variables were investigated. Somewhat surprisingly, few significant associations were detected between expression of miR-21, miR-92a, miR-101, miR-106a and miR-145 and clinicopathological variables, including age, gender, tumor stage, differentiation, localization and specific histomorphologic characteristics such as vascular invasion, perineural infiltration and lymphocyte infiltration (Table 2). MiR-92a and miR-106a were associated with differentiation, as higher median expression levels were found in intermediately differentiated tumors than in well and poorly differentiated tumors (p=0.003 and p=0.01, respectively). Also, some associations were found between miR-31, miR-92a and miR106a expression and tumor localization, as miR-31 exhibited higher expression in colon tumors while miR-92a and miR106a had higher expression levels in rectal tumors (p=0.02, p=0.05 and p=0.05, respectively).

**Figure 2:**
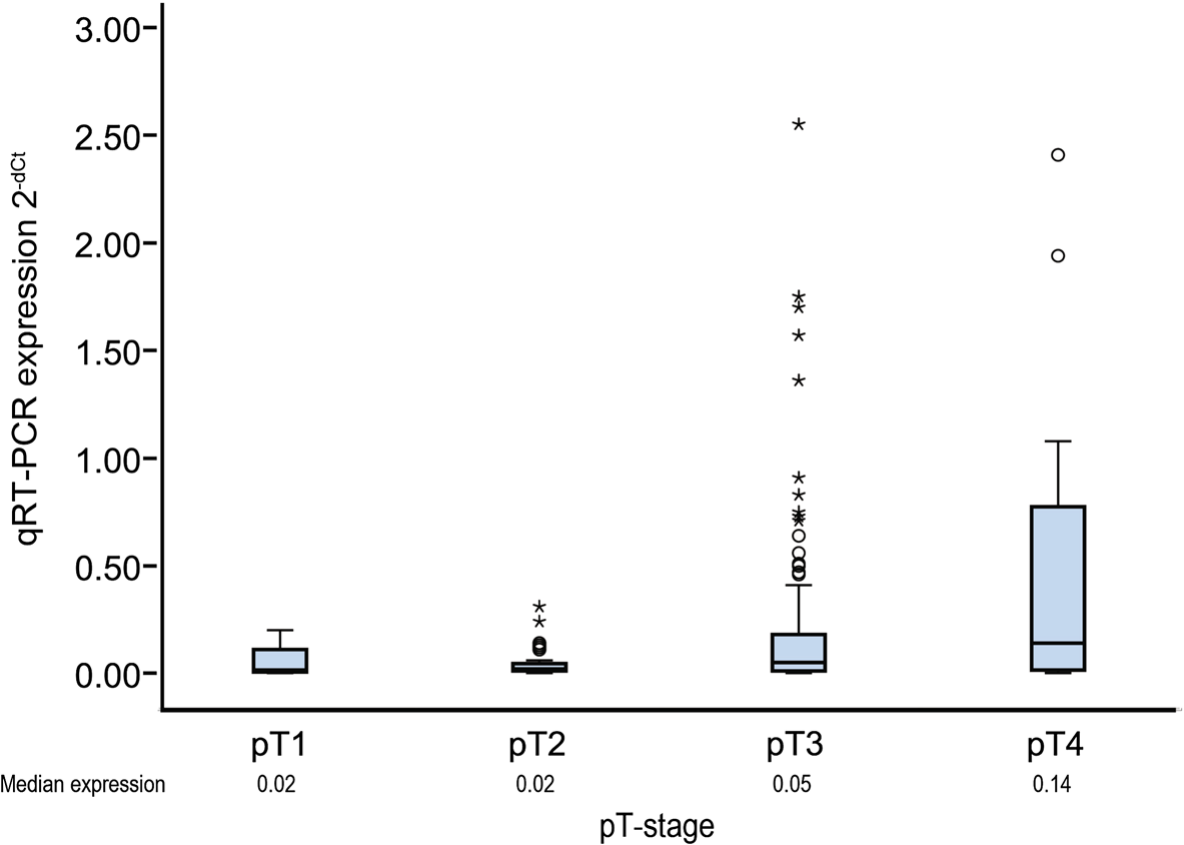
**MiR-31 expression according to pT-stage at diagnosis.** Boxplot showing qRT-PCR relative quantities (using the 2^-dCt^ method) of miR-31 according to pT-stage, indicating that expression of miR-31 increased with increasing pT stage (p=0.004, Kruskal-Wallis test). Circles represent outliers while stars represent extreme outliers.

For miR-31, an association with tumor stage, and in particular with pT stage was found, as relative median expression of miR-31 increased with pT stage (0.015, 0.02, 0.05, and 0.14 for pT1, pT2, pT3, and pT4, respectively; p=0.004, Kruskal-Wallis test) (Figure 2 and Table 2). High miR-31 expression was also associated with poorly differentiated tumors, as relative mean expression was 0.2, 0.04 and 0.02 for poor, intermediate and well differentiated tumors, respectively (p=0.001, Kruskal-Wallis test), which is also in accordance with previous findings [12].

### MiRNA expression and associations with patient outcome

**Figure 3:**
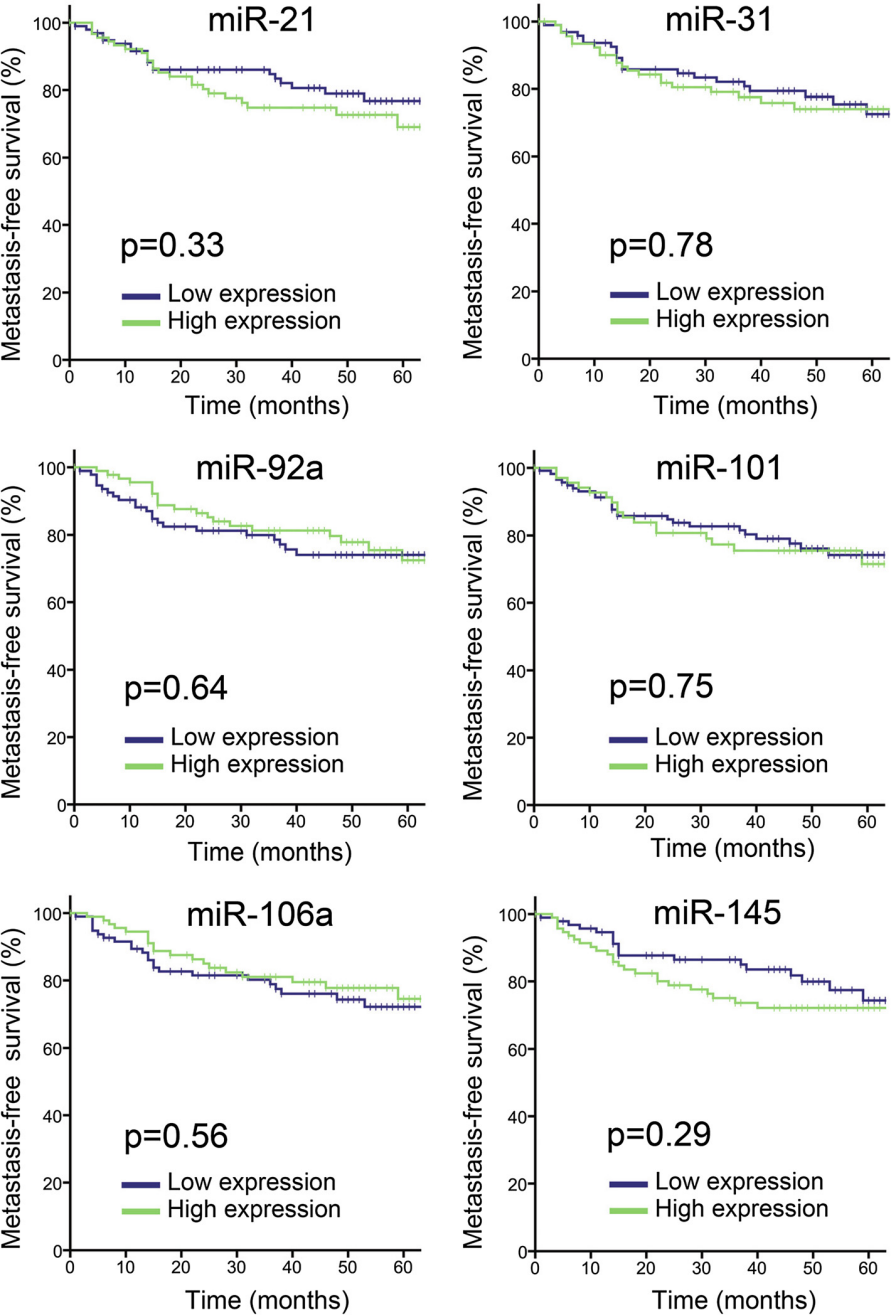
**MiRNA expression and metastasis-free survival.** Kaplan-Meier survival plots of metastasis-free survival for the six selected miRNAs. The 193 patients were divided into low and high expression of the respective miRNA based on the median value (low expression n=97 and high expression n=96).

To analyze associations with outcome, survival was estimated using the Kaplan-Meier method and compared using the log-rank test. As there are no generally recognized cut-off values for the miRNAs analyzed in this work, different values were explored to arrange data (including mean, median and tertiles). Regardless of the cut-off value used, we found no significant associations between expression of any of the analyzed miRNAs and metastasis-free (Figure 3) or overall survival. Similar results were obtained using univariate Cox regression analysis with miRNA expression levels as continuous variables (data not shown).

## Discussion

Although miR-31 was expressed at relatively low levels compared with some of the other candidates, high expression was associated with advanced tumor stage at diagnosis, and particularly with pT-stage, in accordance with previous results [9,10]. There are multiple predicted targets for miR-31, but few have been fully validated at present. One proposed target of miR-31 that has been experimentally investigated is Special AT-rich Binding protein 2 (SATB2), which is involved in transcriptional regulation and chromatin remodeling [18]. In an immunohistochemical study performed in 146 colorectal tumors, low expression of SATB2 was associated with metastasis development and poor prognosis [19]. Another target that has been shown to be regulated by miR-31 is the T lymphoma Invasion And Metastasis gene 1 (TIAM1), which is a guanidine exchange factor for Rac GTPase and when over-expressed, it prevents TGF-β and TNF-α dependent motility and invasion in CRC cell lines [20]. The postulated effects of miR-31 on SATB2 and TIAM1 are consistent with the associations between miR-31 expression and advanced tumor stage, observed by us and others, but clearly, the regulatory activity of miR-31 is still incompletely understood in CRC.

MiR-92a was included in the analyses because it has been proposed as an early-detection biomarker in plasma and stool [13,21]. In general, one would expect an early-detection biomarker to be ubiquitously expressed in the tissue of interest, and although several tumors in our study had relatively high levels of miR-92a, low levels were found in a substantial proportion of the samples. Also, over-expression of miR-92a has been found in other cancer types, such as hepatocellular carcinoma and leukemia [22,23], which suggest that further evaluation is necessary to determine its specificity and sensitivity as an early-detection biomarker. Although miR-92a was not primarily included in this study for its prognostic relevance, it was recently proposed as a key oncogenic component of the miR-17-92 cluster through targeting and down-regulating the proapoptotic protein Bim in CRC, suggesting that the functional role of miR-92a in CRC should be further elucidated [24].

MiR-21 is one of the more extensively studied miRNAs in CRC and was included in our study because of its proposed association with advanced tumor stage and outcome in CRC [12,14]. In the present work, miR-21 exhibited the highest relative expression and the widest expression range of the examined candidates, but no significant associations with clinicopathological data or outcome were found. Although some investigators have identified this miRNA as clinically relevant, other exploratory studies of miRNA expression in CRC have not been able to verify these findings [25-27]. It has been speculated that discrepancies might be explained by the composition of patient cohorts, particularly regarding tumor localization, as the association between miR-21 and survival has primarily been documented in colon cancer [28]. However, in our cohort no differences were found when comparing the clinical relevance of miR-21 expression in colon and rectum cancer. In most of the previous studies, miR-21 expression was reported relative to paired normal tissues, whereas only tumor tissue was available from our patients, which might influence interpretation of results [17]. However, among the reports that did not identify miR-21 as relevant for outcome in CRC, both analysis of tumor tissue alone and paired tumor and normal samples were used, suggesting that this may not be the only explanation for the discrepancies.

When the primary objective is to identify cancer specific molecules, the inclusion of normal tissues is necessary, whereas, in the current project the aim was to evaluate previously identified potential biomarkers, which is a different setting. Importantly, normal tissue is often not obtainable for analysis, and expression of molecular targets in normal tissues might vary considerably between patients, and not necessarily in concert with the corresponding tumor sample. Thus, it is probably both practicable and necessary to develop assays that are independent of normal tissue. Another related challenge concerns the definition of biologically relevant cut-off levels, which have not been determined for specific miRNA in different tissues. We explored multiple cut-off levels, but associations with clinicopathological parameters and outcome for all the candidates remained relatively similar.

MiR-101, miR-145 and miR-106a have previously been associated with cancer-relevant biological processes, such as growth, proliferation and inhibition of apoptosis, or with clinical outcome in CRC [7-9], but few associations with clinicopathological parameters or outcome were found in our cohort. MiR-101 was hardly detectable in tumor samples, which is in accordance with its proposed function as a tumor suppressor that is lost during tumorigenesis. Interesting recent findings in pancreatic cancer suggest miR-101 as a key regulator of stem cell protein markers; its loss favoring the stem cell phenotype and its re-expression constituting a possible therapeutic strategy [29]. Down-regulation of miR-145 was also identified as an early event in CRC carcinogenesis, which might explain why associations with clinical variables in invasive tumors were absent in our tumor panel. The biological relevance of miR-145 in CRC has, however, been repeatedly confirmed, and this miRNA is also being explored as a therapeutic target [30,31]. MiR-106a was in a recent review identified as consistently up-regulated in CRC (relative to normal colon) which would be in agreement with our findings [32]. It has also been identified in stool samples in CRC patients, and has been suggested as an early detection biomarker [33], but even if extensively studied in several cancer forms, its function and clinical relevance remain unclear.

## Conclusions

It has become evident over the last decade that miRNAs contribute to the pathogenesis of a broad variety of human disease, including cancer. Their relatively small number combined with large potential downstream regulatory effects and unique chemical stability make these molecules interesting biomarker candidates. Although the miRNAs analyzed in the present study were chosen on the basis of biomarker potential and biological relevance in CRC, major clinical significance could only be confirmed for miR-31 in our study cohort. It seems clear that the role of miRNAs as colorectal cancer biomarkers is still undetermined, emphasizing the need for further investigations in the exploratory setting and to validate potential biomarkers.

## Competing interests

The authors declare that they have no competing interests.

## Authors’ contributions

KS carried out the qRT-PCR and normalization, performed parts of the statistical analysis and drafted the manuscript. KB performed the main statistical analysis. TWA carried out the tissue preparation and RNA isolation. ØF contributed with critical revisions of the manuscript. KF participated in the study design and coordination and helped draft the manuscript. All authors have read and approved the final manuscript.

## Pre-publication history

The pre-publication history for this paper can be accessed here:

http://www.biomedcentral.com/1471-2407/12/505/prepub
